# Light, Dyes, and Action: Photodynamic Inactivation of *Leishmania amazonensis* Using Methylene Blue, New Methylene Blue, and Novel Ruthenium-Based Derivatives

**DOI:** 10.3390/biology14121710

**Published:** 2025-11-30

**Authors:** Deyvison Rhuan Vasco-dos-Santos, Natália Vacani-Martins, Fabrício Cordeiro Moreira da Silva, Luiz Anastácio Alves, Zênis Novais da Rocha, Andrea Henriques-Pons, Eduardo Caio Torres-Santos, Marcos André Vannier-Santos

**Affiliations:** 1 Laboratório de Inovação em Terapias, Ensino e Bioprodutos, Instituto Oswaldo Cruz (LITEB—IOC), Fundação Oswaldo Cruz (Fiocruz), Rio de Janeiro 21040-900, RJ, Brazil; natalia.vacani1989@gmail.com (N.V.-M.); andreah@ioc.fiocruz.br (A.H.-P.); 2 Laboratório de Comunicação Celular, Instituto Oswaldo Cruz (LCC—IOC), Fundação Oswaldo Cruz (Fiocruz), Rio de Janeiro 21040-900, RJ, Brazil; fabriciocmoreira@gmail.com (F.C.M.d.S.); alveslaa@ioc.fiocruz.br (L.A.A.); 3 Instituto de Química, Universidade Federal da Bahia (UFBA), Campus Universitário de Ondina, Salvador 40170-290, BA, Brazil; zenis@ufba.br; 4 Laboratório de Bioquímica de Tripanossomatídeos, Instituto Oswaldo Cruz (LBqT—IOC), Fundação Oswaldo Cruz (Fiocruz), Rio de Janeiro 21040-900, RJ, Brazil; ects@ioc.fiocruz.br

**Keywords:** neglected tropical diseases, cutaneous leishmaniasis, photodynamic therapy, cell death, apoptosis

## Abstract

Photodynamic therapy (PDT), which combines light and dyes, has emerged as a cost-effective, selective, and less toxic alternative to conventional chemotherapy for cutaneous leishmaniasis (CL), offering potential benefits for millions, especially those who are socioeconomically vulnerable. Therefore, this study investigated the in vitro effects of methylene blue (MB), a widely used photosensitizer with proven clinical efficacy, along with its derivatives—new methylene blue—NMB, NMB-B, and NMB-P—in PDT against *L. amazonensis* promastigotes, using a red LED device. Inhibitory concentrations (ICs) and 168 h proliferation curves were obtained. The production of reactive oxygen species (ROS) and the mechanism of cell death induction were analyzed by flow cytometry. PDT enhanced leishmanicidal effects compared to non-PDT conditions, reducing ICs by up to 85% and outperforming miltefosine, reaching the submicromolar range (IC_25NMB-P_ = 0.73 ± 0.16 µM, *p* < 0.05). The proliferation curve showed a consistent inhibitory effect, with MB exhibiting a greater decline than miltefosine, a pattern also observed with MB derivatives. PDT also increased ROS production by up to 5-fold and induced apoptosis-like cell death, characterized by AV^+^ parasites (up to 51.49 ± 2.90%, *p* < 0.0001). The results demonstrated that the tested dyes effectively eliminated *L. amazonensis* promastigotes, highlighting the potential of the NMB derivatives as photosensitizers and supporting further investigations.

## Simple Summary

Light has been used to treat diseases since ancient Egypt. Today, when combined with clinically safe dyes, it forms the basis of a low-cost, non-toxic, and effective treatment called photodynamic therapy, employed in the treatment of cutaneous leishmaniasis (CL). The disease is caused by *Leishmania* parasites and transmitted by sand flies, affecting millions of people worldwide. However, the current treatments are often limited by unresponsive cases and toxicity to the liver, kidneys, and heart. Seeking more effective alternatives, this study explores whether a well-known dye (methylene blue—MB) and its derivatives (new methylene blue—NMB, NMB-B and NMB-P), when combined with light, could eliminate *L. amazonensis*, a parasite that causes CL. Our results showed that these dyes, under light exposure, eliminated the parasites more effectively than the reference drug miltefosine. Additionally, the compounds increased the production of antiparasitic substances, causing severe damage and parasite death. This study demonstrated that the compounds, when combined with light, have great therapeutic potential, offering new perspectives for more effective treatments.

## 1. Introduction

The “magic bullet” concept, introduced by Paul Ehrlich in 1906, posited that therapeutic agents could selectively eliminate pathogens without harming host cells, establishing the conceptual framework for chemotherapy [1,2]. His studies on dyes, including methylene blue (MB) for malaria and trypan red for trypanosomiasis, culminated in the discovery of salvarsan, the first effective chemotherapeutic agent for syphilis [3,4,5,6]. This approach encouraged researchers to investigate new treatments [7], including Gaspar Vianna, who in 1912 demonstrated the efficacy of emetic tartar [antimony potassium tartrate, Sb(III)] for cutaneous (CL) and mucosal leishmaniasis in Brazilian patients from the Amazon region, Minas Gerais, and Bahia. He also documented its associated adverse effects, such as arthralgia, headache, emesis, and nephritis [8,9].

Pentavalent antimonials [Sb(V)] were introduced in the 1940s to mitigate the pronounced toxic of Sb(III) [10,11]. Nevertheless, Sb(V) exhibits toxicity effects on the nervous system [12], DNA [13], cochlea [14], pancreas [15], liver [16,17], kidneys [18], and heart [19,20], with reports of treatment-related fatalities [21,22]. Despite its severe adverse effects, parenteral administration and resistance selection, Sb(V) remains the first-line therapy for CL, the most prevalent clinical form of leishmaniasis [23,24,25,26]. CL is a highly neglected condition that affects nearly one million people annually, manifesting as skin lesions at the site of the sand fly hematophagy [27,28,29,30]. These lesions may progress to facial disfigurement, leading to social stigma and psychological distress [31,32], including suicidal ideation. For these reasons, CL has been referred to as the “mark of shame” [33].

Consequently, individuals affected by CL face not only the disease per se but also its treatment, characterized by substantial physical suffering, as consistently documented in patient accounts [34,35,36,37]. Thus, novel therapeutic approaches are urgently required, as currently repurposed chemotherapeutic agents, such as pentamidine, paromomycin, amphotericin B, and miltefosine, remain limited by toxicity, resistance selection, and elevated costs [38,39,40]. This need is particularly critical given the complexity of leishmaniasis and its strong association with socioeconomically disadvantaged populations [41,42,43].

Promising therapeutic strategies include drug combinations [44,45], peptides [46,47], immunotherapy [48,49], and photodynamic therapy (PDT), a low-toxicity, non-invasive, and accessible photochemotherapy [50,51]. PDT relies on the interaction of a photosensitizer (PS), light, and molecular oxygen to induce oxidative stress, resulting in cellular damage and death [52,53]. Its clinical efficacy against CL has been demonstrated through the use of light-emitting diode (LED) or natural light, leading to complete lesion resolution without recurrence or severe adverse effects [54,55].

Among available PSs, MB stands out for its strong light absorption within the therapeutic window, well-characterized photochemistry, capacity to damage biomolecules, and proven efficacy in inducing cell death in target organisms [56]. Its low cost, high hydrosolubility, and suitability for topical application may contribute to shortening treatment durations and reducing systemic toxicity [57,58]. MB use in PDT has demonstrated efficacy against a range of diseases, including onychomycosis [59], encephalopathy [60], oral candidiasis [61], pityriasis versicolor [62], and trichomoniasis [63], as well as against *Trypanosoma cruzi*, the etiological agent of Chagas disease [64]. MB was also proven active against the CL caused by *Leishmania amazonensis* in murine [65] and hamster models [66], as well as humans [67]. Furthermore, the use of repositioned compounds, such as MB, is associated with greater translational potential, as drug repurposing reduces failure risk, development time, and costs, compared to traditional discovery approaches [68].

Our group recently demonstrated the in vitro activity of MB, new methylene blue (NMB), and its novel ruthenium-based derivatives, new methylene blue B (NMB-B) and new methylene blue P (NMB-P), against *L. amazonensis* [69]. The compounds showed a high selectivity and submicromolar potency, inducing parasite death through mechanisms resembling apoptosis and necrosis, associated with increased reactive oxygen species (ROS) production and a decline in mitochondrial membrane potential. In this study, we investigated the PDT-mediated effects of these dyes on *L. amazonensis* promastigotes, hypothesizing that the new derivatives are photosensitive and that the tested compounds exhibit enhanced leishmanicidal activity in vitro under light activation.

## 2. Materials and Methods

### 2.1. Compounds and Parasite Culture

The MB, NMB, miltefosine, and reagents used for the synthesis of NMB-B and NMB-P—[RuCl_3_·nH_2_O, 2,2′-bipyridine (bpy) and 1,10-phenanthroline (phen)]—were acquired from Sigma-Aldrich (St. Louis, MO, USA), as previously described [69]. All compounds were dissolved in phosphate-buffered saline (PBS; Gibco—Grand Island, NY, USA), except for the miltefosine, which was prepared in sterile water. Solutions were stored at −20 °C. Promastigote forms of *L. amazonensis* (IFLA/BR/1967/PH8) were obtained from the Instituto Oswaldo Cruz *Leishmania* collection (CLIOC/Fiocruz, Rio de Janeiro, RJ, Brazil) and maintained at 26 °C in Schneider’s Insect Medium (Sigma), supplemented with 20% fetal bovine serum (FBS; Campinas, SP, Brazil) and 1 μL/mL of penicillin/streptomycin (Sigma).

### 2.2. Photodynamic Inhibition Assay

Promastigote forms of *L. amazonensis* (10^6^ parasites/mL) were plated in 96-well flat-bottom microplates and treated with 2-fold serial dilutions of the compounds, starting from their previously determined IC_50_ values in the absence of PDT. After a 10 min incubation to allow the PS’s uptake [70,71], parasites were irradiated using a red LED device with a mean intensity of 1927.0 ± 35.5 μW/cm^2^, measured with a portable digital photoradiometer (HD2302.0, Dellt, Santo André, SP, Brazil). Irradiation was performed for 60 (1.16 × 10^−4^ J/cm^2^), 300 (5.78 × 10^−4^ J/cm^2^) or 600 s (1.16 × 10^−3^ J/cm^2^). Each well was individually irradiated from the bottom upward, with the light source placed in direct contact with the microplate. A mask was used to cover the control wells from light exposure.

After 24 h of incubation, parasite viability was estimated using the Alamar Blue assay (Invitrogen, Eugene, OR, USA) [72]. The data were used to calculate the 25%, 50%, and 75% inhibitory concentration values (IC_25, 50, 75_). Additionally, growth curves were generated to evaluate the viability of promastigotes over 168 h following exposure to the compounds at their IC_50_ and PDT. The analysis was performed using a spectrophotometer (Molecular Devices, San Jose, CA, USA) set to 560/590 nm. Wells containing only medium were used as blanks; irradiated but untreated parasites were used as light controls (LED-only); and non-irradiated, untreated parasites were used as negative controls. Miltefosine was used as a positive control.

### 2.3. Flow Cytometry Analysis

Flow cytometric analysis of *L. amazonensis* was conducted using a Cytoflex S flow cytometer (Beckman Coulter, Brea, CA, USA) at the Flow Cytometry Facility—Unit of Multiparametric Analysis of Instituto Oswaldo Cruz. Data were analyzed using CytExpert software, version 2.5 (Beckman Coulter, Brea, CA, USA). We previously reported that promastigotes treated with the tested dyes showed no fluorescence in the channels used in this work [69]. Parasites were subsequently exposed to the IC_50_ of each compound without light exposure, incubated for 10 min, and then irradiated with red LEDs for 300 s (5.78 × 10^−4^ J/cm^2^). After 24 h, a flow cytometric analysis was performed, acquiring a total of 10,000 events within the LP (low population) and HP (high population) regions, corresponding to mostly dead or viable parasites, respectively, as previously described [69].

For ROS production measurements, parasites were labeled with 20 µM 2′,7′-dichlorodihydrofluorescein diacetate (H_2_DCFDA; PROC9, Canoas, RS, Brazil), and antimycin A (AA; 10 µM, Sigma) was used as a positive control. The variation index of ROS (*VI*_ROS_) was calculated as the ratio of the median fluorescence intensity (MFI) of H_2_DCFDA in the 525/40 nm channel for the treated parasites to the MFI of the negative control (MT_H2DCFDA_/MC_H2DCFDA_). For cell death analysis, promastigotes were labeled with FITC Annexin V Apoptosis Detection Kit I (BD Pharmingen, Franklin Lakes, NJ, USA), according to the instructions provided by the manufacturer. Live and heat-killed (60 °C for 15 min) parasites were used as negative and positive controls, respectively. Apoptotic or necrotic events were analyzed based on Annexin V (AV) and propidium iodide (PI) positivity in the 525/40 nm and 585/42 nm channels, respectively.

### 2.4. Statistical Analysis

Assays were performed in triplicate and independently repeated at least twice. Data were plotted using Microsoft Excel (v.2406), and statistical analyses were carried out in GraphPad Prism (v.8; GraphPad Software Inc., San Diego, CA, USA). Data normality was assessed using the Shapiro–Wilk test. Upon confirmation of normal distribution, statistical significance was evaluated using one-way ANOVA, followed by Dunnett’s test for multiple comparisons. For comparisons between two groups, an unpaired *t*-test was used.

## 3. Results

### 3.1. Photodynamic Therapy Enhances Antileishmanial Efficacy of Tested Dyes

**Figure 1:**
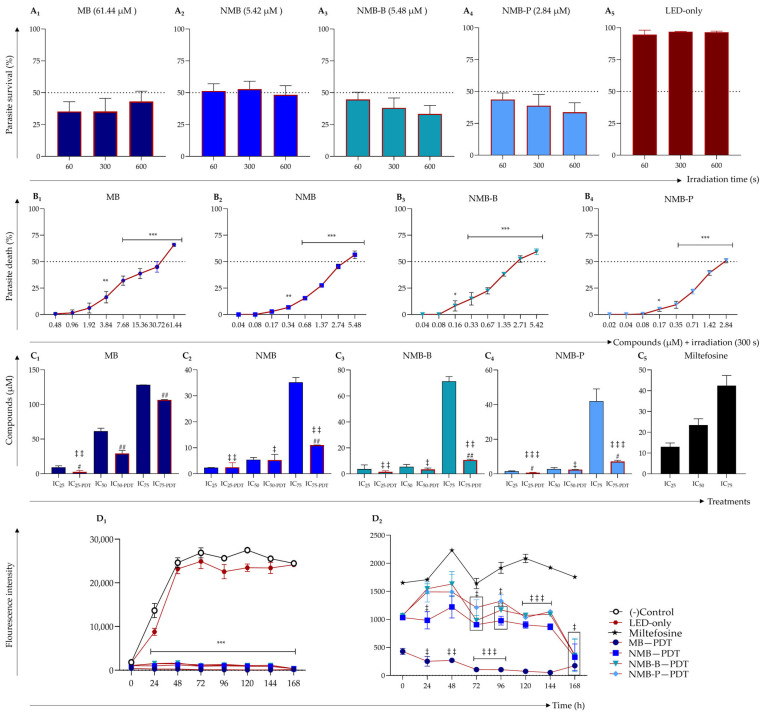
In vitro evaluation of photodynamic therapy (PDT) using methylene blue (MB), new methylene blue (NMB), new methylene blue B (NMB-B), and new methylene blue P (NMB-P), compared to miltefosine, against *Leishmania amazonensis* PH8 strain promastigotes (10^6^ parasites/mL). **Panel A** (**A_1_**–**A_5_**): Optimization of red light-emitting diode (LED) irradiation time for parasites treated with the IC_50_ of MB (**A_1_**—61.44 μM), NMB (**A_2_**—5.42 μM), NMB-B (**A_3_**—5.48 μM) and NMB-P (**A_4_**—2.84 μM); **A_5_** shows untreated parasites exposed to red LED only. Irradiation times were 60 (1.16 × 10^−4^ J/cm^2^), 300 (5.78 × 10^−4^ J/cm^2^) and 600 s (1.16 × 10^−3^ J/cm^2^). **Panel B** (**B_1_**–**B_4_**): Dose-response curves for MB (**B_1_**), NMB (**B_2_**), NMB-B (**B_3_**) and NMB-P (**B_4_**) following PDT (300 s). **Panel C** (**C_1_**–**C_5_**): IC_25_, _50_, _75_ values of MB (**C_1_**), NMB (**C_2_**), NMB-B (**C_3_**) and NMB-P (**C_4_**) with and without PDT (300 s); **C_5_** shows miltefosine values. **Panel D** (**D_1_**,**D_2_**): Parasite proliferation over 168 h. (**D_1_**): Fluorescence intensity among treated, untreated, and LED-only parasites. (**D_2_**): Fluorescence intensity comparison between treated parasites and miltefosine. Data are presented as mean ± SD. (*) *p* < 0.05; (**) *p* < 0.001; (***) *p* < 0.0001 compared to untreated parasites (one-way ANOVA and Dunnett’s post-test). (#) *p* < 0.05; (##) *p* < 0.001 compared to IC_25_, _50_, _75_ values with and without PDT (unpaired *t*-test). (‡) *p* < 0.05; (‡‡) *p* < 0.001; (‡‡‡) *p* < 0.0001 compared to miltefosine (one-way ANOVA and Dunnett’s post-test). IC_25_, IC_50_ and IC_75_ represent the compound concentrations required to inhibit 25%, 50% (half maximal) and 75% of parasite viability, respectively.

*L. amazonensis* promastigotes were exposed to the previously established IC_50_ of MB, NMB, NMB-B, and NMB-P in the absence of PDT (Table 1). Subsequent red LED irradiation was applied for 60, 300 and 600 s. No significant differences were observed among these conditions (Figure 1A_1_–A_4_), although all treatments resulted in a marked reduction in viability (*p* < 0.001) compared to untreated parasites. The LED-only group exhibited a high survival rate (94.57 ± 6.53%) with no significant difference compared to the control (Figure 1A_5_), indicating that LED exposure alone does not compromise promastigote viability. Therefore, a 300 s irradiation time (5.78 × 10^−4^ J/cm^2^) was selected for subsequent assays.

The combination of red LED irradiation with the compounds resulted in a highly significant (*p* < 0.0001), dose-dependent reduction in *Leishmania* promastigote survival compared to the negative control (Figure 1B_1_–B_4_). NMB-B and NMB-P demonstrated the most potent activity, with significant decreases (*p* < 0.05) in viability observed at concentrations as low as 0.16 and 0.17 μM, respectively. The IC_25_, _50_, _75_ values obtained under PDT are presented in Table 1. PDT markedly enhanced the anti-*Leishmania* efficacy of all the tested dyes, resulting in significantly lower inhibitory concentrations compared to those under non-PDT conditions (*p* < 0.05, Figure 1C_1_–C_4_). Reductions reached up to 70.93% for IC_25_ (*p* < 0.05), 52.27% for IC_50_ (*p* < 0.001), and 85.00% for IC_75_ (*p* < 0.001). NMB derivatives exhibited the most pronounced IC_75_ reductions (NMB-B = 10.70 ± 0.58 μM and NMB-P = 7.20 ± 0.68 μM), which were significantly lower than their non-photostimulated values (NMB-B = 71.32 ± 3.56 μM and NMB-P = 37.76 ± 8.91 μM, *p* < 0.05, Figure 1C_3_,C_4_). Likewise, MB and NMB showed significant (*p* < 0.001) IC_75_ reductions (Figure 1C_1_,C_2_).

Compared to miltefosine, PDT-activated dyes significantly (*p* < 0.05) reduced inhibitory concentrations. IC_25_ values decreased between 4- and 17-fold, with NMB-P reaching submicromolar potency (0.73 ± 0.16 µM, *p* < 0.0001). IC_50_ values were also significantly reduced (*p* < 0.05). NMB-P showed the greatest decrease (2.48 ± 0.04 µM), up to 9-fold lower than miltefosine, followed by NMB-B (3.48 ± 0.99 µM) and NMB (5.28 ± 2.19 µM), both achieving reductions greater than 4-fold in comparison to the reference drug. Regarding IC_75_, NMB-P and NMB-B demonstrated the most substantial reductions, reaching 7.20 ± 0.68 µM and 10.70 ± 0.58 µM, respectively. Both values were significantly (*p* < 0.001) lower than miltefosine. These results are further illustrated in Figure 1C_3_,C_4_, which clearly depict the marked decrease in IC_75_ under PDT. Overall, NMB-P emerged as the most promising candidate, consistently demonstrating the lowest IC_25_, _50_, _75_ values.

### 3.2. Dyes Activated by PDT Outperform Miltefosine in Sustained Leishmanicidal Activity

The 168-h proliferation assay revealed no significant difference in viability between LED-exposed and untreated parasites, with both groups maintaining high survival rates (Figure 1D_1_). In contrast, PDT-activated dyes significantly reduced parasite proliferation compared to the reference drug (*p* < 0.05, Figure 1D_2_). MB exhibited the strongest effect, achieving near-total parasite elimination within 24 h. NMB, NMB-B and NMB-P displayed a similar reduction pattern, with a pronounced decline in viability starting at 96 h, ultimately reaching levels lower than those observed for miltefosine and comparable to MB at 168 h.

Additionally, the miltefosine proliferation curve exhibits greater fluctuations, suggesting the presence of parasite populations displaying distinct susceptibility levels, unlike the pattern observed with PDT-associated compounds. Notably, MB maintains consistent suppressive effects. Although NMB, NMB-B and NMB-P show minor viability peaks, they induce a more pronounced and sustained decline in parasite viability compared to miltefosine (Figure 1D_2_).

### 3.3. Ruthenium-Based Photosensitizers Boost ROS Generation

**Figure 2:**
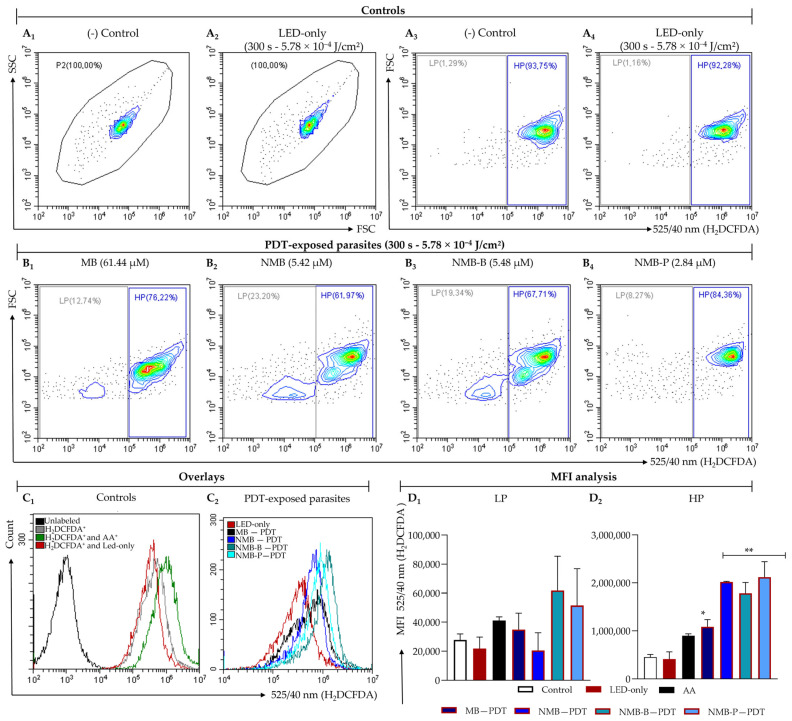
Evaluation of reactive oxygen species (ROS) production by flow cytometry on promastigote forms of the *Leishmania amazonensis* PH8 strain (5 × 10^6^ parasites/mL) exposed to the IC_50_ of methylene blue (MB), new methylene blue (NMB), new methylene blue B (NMB-B) and new methylene blue P (NMB-P), followed by photodynamic therapy (PDT) with red light-emitting diode (LED) irradiation (300 s, 5.78 × 10^−4^ J/cm^2^) and 24 h of incubation. **Panel A** (**A_1_**–**A_4_**): Morphology (**A_1_**,**A_2_**) and percentage of H_2_DCFDA^+^ parasites (**A_3_**,**A_4_**) in control and LED-only groups. **Panel B** (**B_1_**–**B_4_**): Percentage of H_2_DCFDA^+^ parasites exposed to PDT using MB (**B_1_**), NMB (**B_2_**), NMB-B (**B_3_**), and NMB-P (**B_4_**). **Panel C** (**C_1_**_,_**C_2_**): Overlay of fluorescence intensity for control groups (**C_1_**) and PDT-exposed parasites (**C_2_**). **Panel D** (**D_1_**,**D_2_**): Quantification of the MFI for control and treated parasites, separated by subpopulation: LP (**D_1_**) and HP (**D_2_**). Data are presented as mean ± SD. (*) *p* < 0.05; (**) *p* < 0.001 when compared to negative control by one-way ANOVA and Dunnett’s post-test. SSC = side scatter; FSC = forward scatter; 525/40 nm = fluorescence channel; H_2_DCFDA = 2′,7′-dichlorodihydrofluorescein diacetate; AA = antimycin A; MFI = median fluorescence intensity; LP = low population; HP = high population; IC_50_ = 50% maximal inhibitory concentration.

ROS analysis revealed no difference in the percentage of H_2_DCFDA^+^ events between the negative control and LED-only group, both showing approximately 84.14 ± 0.47% of parasites in the HP region (Figure 2A_3_,A_4_). A similar pattern was observed in the overlay (Figure 2C_1_). In contrast, promastigotes subjected to PDT exhibited an increased H_2_DCFDA fluorescence intensity (Figure 2C_2_), followed by a 10-fold decrease in the HP region and up to a 7-fold increase in the LP region (Figure 2B_1_–B_4_).

The *VI*_ROS_ in the LED group did not differ significantly from the control across any subpopulation (Table 2). However, the tested dyes induced a significant increase in ROS levels (*p* < 0.05), with HP exhibiting a 5-fold elevation (*p* < 0.001, Figure 2D_2_). Interestingly, NMB (4.49 ± 0.53), NMB-P (4.67 ± 0.13), and NMB-B (4.00 ± 1.00) surpassed the *VI*_ROS_ value observed for AA (1.99 ± 0.16). In the LP region, the novel ruthenium-complexed derivatives, NMB-B and NMB-P, also demonstrated efficacy, doubling ROS production relative to the control. This reinforces their capacity to induce oxidative stress in *L. amazonensis* under PDT.

### 3.4. Photodynamic Therapy Triggers Extensive Cell Death in Leishmania

**Figure 3:**
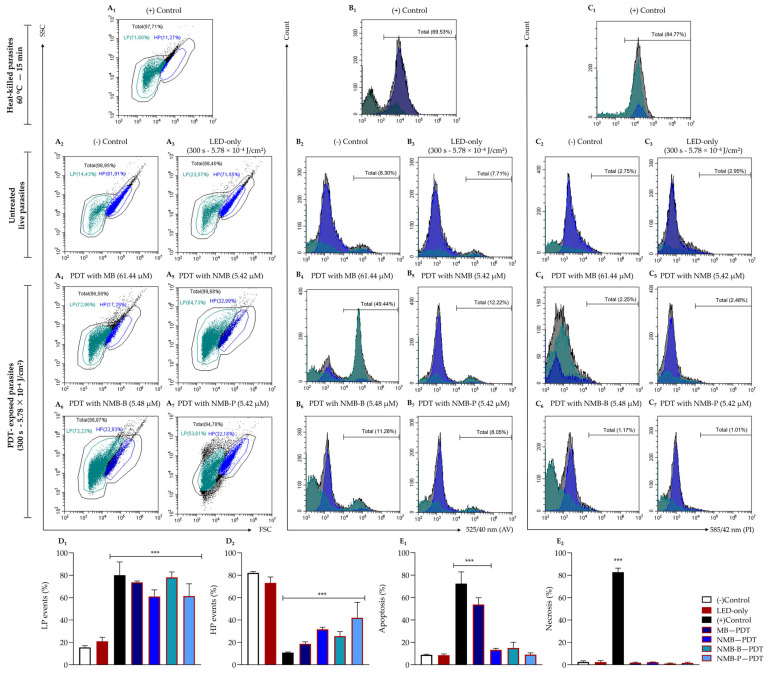
Cell death evaluation by flow cytometry on promastigote forms of the *Leishmania amazonensis* PH8 strain (10^6^ parasites/mL) treated with the IC_50_ of methylene blue (MB), new methylene blue (NMB), new methylene blue B (NMB-B) and new methylene blue P (NMB-P), followed by photodynamic therapy (PDT) using red light-emitting diode (LED) irradiation (300 s, 5.78 × 10^−4^ J/cm^2^) and incubation for 24 h. Parasites were labeled with AV and PI. Heat-killed parasites (60 °C, 15 min) were used as the positive control, while untreated parasites were used as the negative control. The LED-only group represents untreated parasites exposed to irradiation. **Panel A** (**A_1_**–**A_7_**): Dot plots showing the FSC/SSC-based morphology of positive (**A_1_**) and negative controls (**A_2_**), LED-only group (**A_3_**), and PDT-exposed parasites with MB (**A_4_**), NMB (**A_5_**), NMB-B (**A_6_**) and NMB-P (**A_7_**). **Panel B** (**B_1_**–**B_7_**): Histograms of AV fluorescence intensity in positive (**B_1_**) and negative controls (**B_2_**), LED-only group (**B_3_**), and PDT-exposed parasites with MB (**B_4_**), NMB (**B_5_**), NMB-B (**B_6_**) and NMB-P (**B_7_**). **Panel C** (**C_1_**–**C_7_**): Histograms of PI fluorescence intensity in positive (**C_1_**) and negative control (**C_2_**), LED-only group (**C_3_**), and PDT-exposed parasites with MB (**C_4_**), NMB (**C_5_**), NMB-B (**C_6_**) and NMB-P (**C_7_**). **Panel D** (**D_1_**,**D_2_**): Percentage of parasites within LP (**D_1_**) and HP (**D_2_**) regions. **Panel E** (**E_1_**,**E_2_**): Percentage of apoptotic (**E_1_**) and necrotic events (**E_2_**). Data are presented as mean ± SD. (***) *p* < 0.0001 when compared to control by one-way ANOVA and Dunnett’s post-test. SSC = side scatter; FSC = forward scatter; 525/40 nm and 585/42 nm = fluorescence channels; AV = annexin V; PI = propidium iodide; LP = low population; HP = high population; IC_50_ = 50% maximal inhibitory concentration.

The cell death analysis revealed similar morphological profiles between the negative control and the LED-only group, characterized by well-defined HP and LP regions (Figure 3A_2_,A_3_). Most events were concentrated within the HP region (77.63 ± 6.30%), with forward and side scatter (FSC and SSC) values around 10^5^, indicative of preserved parasite integrity. A minor fraction (16.98 ± 1.97%) was in the LP region, suggesting high viability, as indicated by a low AV (8.71 ± 0.24%, Figure 3B_2_,B_3_) and PI (2.41 ± 0.04%, Figure 3C_2_,C_3_) positivity. These findings confirm that LED exposure alone does not compromise parasite viability, as demonstrated by the irradiation time optimization (Figure 1A_5_) and proliferation (Figure 1D_1_) assays. In contrast, heat-killed parasites exhibited a significant shift (*p* < 0.0001) in subpopulation distribution compared to the negative control, with a 71% decrease in the HP and a 50% increase in the LP (Figure 3A_1_). This effect correlated with elevated AV (72.44 ± 10.52%, *p* < 0.0001, Figure 3B_1_) and PI (82.78 ± 3.63%, *p* < 0.0001, Figure 3C_1_) positivity.

Parasites exposed to PDT displayed a profile comparable to the positive control, characterized by a highly significant shift (*p* < 0.0001) from HP to LP, resulting in a 62% increase in the LP region (Figure 3D_1_). Marked morphological alterations were observed, including >2-log reductions in both FSC and SSC parameters (Figure 3A_4_–A_7_), suggesting cell fragmentation, cytoplasmic degradation, and organelle loss. Despite extensive morphological damage, AV and PI labeling remained nonsignificant (Figure 3E_1_,E_2_), except for MB, which showed a significant increase in AV^+^ parasites (51.46 ± 5.90%, *p* < 0.0001, Figure 3B_4_), similar to the profile observed in heat-killed parasites.

The absence of PI^+^ parasites may reflect advanced cellular degradation, hindering labeling. In addition, the low percentage of AV^+^ parasites for NMB and its novel derivatives (NMB-B and NMB-P, Figure 3B_5_–B_7_) is likely attributable to the presence of phosphatidylserine in debris with residual membrane integrity, as indicated by low FSC (<10^4^) and reduced SSC values. This effect is probably linked to the high ROS production described (Table 2, Figure 2), suggesting a late-stage apoptosis-like death mechanism.

## 4. Discussion

The therapeutic application of light dates back to ancient civilizations, with a pivotal milestone in 1900 when Oscar Raab, while evaluating the effects of acridine on paramecia during a day of heavy thunderstorms, noted that light exposure enhanced the dye’s toxicity, leading to rapid protozoan elimination [73,74,75,76]. Later, von Tappeiner elucidated the oxygen dependence of this phenomenon and coined the term photodynamic therapy [77,78]. Since then, dyes have become central to PDT, broadening their application to cancer therapy [79,80,81,82] and infections caused by bacteria [83,84], fungi [85], and viruses, including SARS-CoV-2 [86,87], as well as pathogenic kinetoplastids such as *T. cruzi* [88] and *Leishmania* spp. [89]. Among dyes, phenothiazines, particularly MB, stand out due to their low cost, high ROS generation, and demonstrated efficacy in both in vitro and in vivo models [90,91,92,93].

Our findings are consistent with the literature, showing a highly significant increase (*p* < 0.0001) in ROS levels in HP, with MB doubling ROS production and its derivatives (NMB, NMB-B and NMB-P) quadrupling it (Table 2). Moreover, NMB-B and NMB-P doubled ROS levels in LP, an effect not observed in the absence of PDT [69], underscoring their potential as effective PSs. In PDT, ROS are generated through two main mechanisms. In type I, the excited triplet state of the PS facilitates electron transfer, resulting in the formation of superoxide anion, hydrogen peroxide, and hydroxyl radicals. In type II, energy is transferred to molecular oxygen, producing highly cytotoxic singlet oxygen [94,95,96]. Given its short diffusion range (<0.02 µm), singlet oxygen induces localized photodamage at sites where PSs accumulate [97]. MB-mediated PDT induces ROS generation via both type I and type II mechanisms, leading to oxidative damage in the cytosol, lysosomes, and nucleus, with a predominant impact on the mitochondria, thereby initiating the apoptotic cascade [56,57,70,98].

*Leishmania* presents a single mitochondrion that extends throughout the entire cell and plays a central role in ROS generation. Consequently, this organelle is essential for parasite survival and constitutes a key target for leishmanicidal agents that disrupt its structure and function, leading to apoptosis-like cell death [99,100,101]. This process is typically marked by cell shrinkage, rounding, mitochondrial membrane depolarization, chromatin condensation, DNA fragmentation and externalization of phosphatidylserine [102,103], as demonstrated in *Leishmania tropica* [104] and *L. amazonensis* [70] promastigotes under MB-mediated PDT.

Our results provide additional support for this apoptosis-like mechanism, as evidenced by a significant increase in AV^+^ promastigotes (51.49 ± 2.90%, *p* < 0.0001, Figure 3B_4_,E_1_) following MB-mediated PDT, which was four-fold higher than that observed in parasites exposed to MB without photostimulation (11.08 ± 1.05%) [69]. Ozlem-Caliskan et al. [104] reported the presence of apoptotic bodies detected by 4′,6-diamidino-2-phenylindole (DAPI) staining, which might explain the lower AV labeling seen in PDT with NMB, NMB-B and NMB-P (Figure 3B_5_–B_7_), as these structures are indicative of late-stage apoptosis. Nevertheless, the unequivocal formation of apoptotic bodies in *Leishmania* has not been demonstrated, possibly due to its fence-like parallel array of subpellicular microtubules restricting plasma membrane flexibility and thereby hindering the development of these structures [105,106].

Additionally, severe morphological damage was observed in causative agents of CL, including *Leishmania major*, *Leishmania braziliensis* and *L. tropica*, after PDT with different PSs, leading to a loss of morphological distinction between the nucleus and kinetoplast, which became unrecognizable [57,104]. These parasites were described as “ghost cells” [104]. However, despite pronounced intracellular disorganization, the overall cell shape remained partially preserved, presumably due to the high stability of the membrane–microtubule association characteristic of trypanosomatids. Using electron microscopy, Pimenta et al. documented extracellular parasites in which the subpellicular microtubules preserved plasma membrane integrity, even in the absence of discernible organelles [107]. Thus, the formation of canonical apoptotic bodies, typically resulting from membrane blebbing, appears unlikely in *Leishmania*.

This study demonstrated pronounced morphological alterations in *L. amazonensis* promastigotes, with all dyes under PDT inducing a significant shift in events from the HP to LP region. The consequent reduction in PI labeling suggests extensive DNA degradation, considering the markedly higher percentage of PI^+^ parasites observed in the absence of PDT ($\bar{x}$ = 25.70 ± 6.33%) [69], along with evidence indicating that *Leishmania* may become PI-negative shortly after cell death [105]. In a complementary manner, our group recently demonstrated through image cytometry that *L. amazonensis* exposed to MB, NMB, NMB-B, and NMB-P exhibited reduced refringence, cell rounding, cytoplasmic degradation, membrane damage, flagellum loss and presumably DNA fragmentation [69], all consistent with late-stage apoptosis. PDT likely enhanced these effects, contributing to the observed cell death profile.

**Figure 4:**
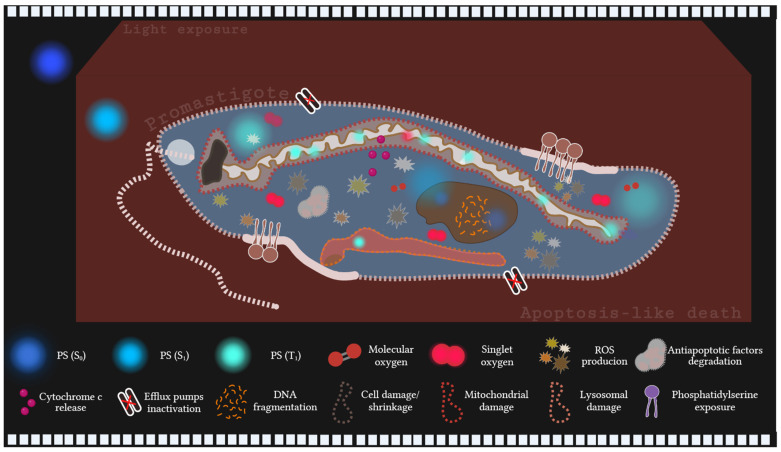
Mechanistic inference model of the photodynamic therapy (PDT) action of methylene blue (MB), new methylene blue (NMB), new methylene blue B (NMB-B) and new methylene blue P (NMB-P) in *Leishmania* promastigotes. These dyes act as photosensitizers (PSs), absorbing light and transitioning from the ground state (S_0_) to the excited singlet state (S_1_), with the potential to reach the triplet state (T_1_). Within promastigotes, PSs accumulate in parasite compartments, including the cytoplasm, lysosomes, mitochondria, and nucleus. In the T_1_ state, PSs generate reactive oxygen species (ROS) via electron transfer, such as superoxide anion (O_2_^−^) and hydrogen peroxide (H_2_O_2_), while energy transfer produces singlet oxygen (^1^O_2_), a highly cytotoxic species. The resulting oxidative stress triggers an apoptotic cascade, characterized by cytochrome c release, the degradation of antiapoptotic factors, and the inactivation of efflux pumps (e.g., aquaporin 1 and P-glycoprotein), thereby enhancing the PSs efficacy. Consequently, structural alterations occur, including cell shrinkage, mitochondrial membrane depolarization, lysosomal damage, DNA fragmentation and phosphatidylserine exposure [56,57,69,70,95,97,98,102,104,108,113,114]. Created in BioRender. Vasco-dos-Santos, D. (2025) https://BioRender.com/po42xqa.

Although the mechanisms of action of MB derivatives are not fully elucidated, Zheng et al. [108] demonstrate that NMB-mediated PDT induces irreversible damage in *Fonsecaea nubica*, causing cell shrinkage and the destruction of organelles, including the nucleus and mitochondria, through ROS, such as singlet oxygen. These findings resemble those described for MB-mediated PDT in *Leishmania*, suggesting that ROS-induced apoptosis comprises a key mechanism of action for NMB and possibly for its derivatives NMB-B and NMB-P (Figure 4). Mitochondria appear to play a central role in this process, as the tested dyes reduced mitochondrial membrane potential in the promastigote and amastigote forms of *L. amazonensis* by up to 41% and 98%, respectively [69].

The superior efficacy of MB derivatives in PDT, as observed by multiple analyses (Table 1 and Table 2, Figure 1, Figure 2 and Figure 3), may be attributed to structural modifications that enhance their interactions with solvents, light and biological tissues, as exemplified by NMB and 1,9 dimethyl-methylene blue (DMMB) [58]. These compounds exhibit greater phototoxicity due to their higher lipophilicity and cationic charge. The IC_50_ value for MB-mediated PDT obtained in this study (29.33 ± 4.31 µM) is comparable to previously reported values for *L. tropica* (20.21 µM) [104] and *L. amazonensis* (20.00 µM) [67]. However, the derivatives demonstrated IC_50_ values 5- to 11-fold lower than those of MB and significantly lower than miltefosine (*p* < 0.05), with NMB-P achieving the submicromolar range (IC_25_ = 0.76 ± 0.16 µM). Phenothiazines also showed superior efficacy against *T. cruzi* amastigotes compared to benznidazole, with NMB reaching an IC_50_ of 0.09 µM [109]. Furthermore, NMB outperformed MB and other PSs in the treatment of burns in mice infected with multidrug-resistant *Acinetobacter baumannii* [110].

Drug resistance to the chemotherapeutic agents used in CL treatment [e.g., Sb(V), amphotericin B, pentamidine, paromomycin and miltefosine] poses a major challenge, primarily attributed to efflux mechanisms mediated by ATP-binding cassette (ABC) transporters, including aquaporin 1 and P-glycoprotein [24,111,112]. Thus, PDT emerges as a promising strategy, since mitochondria-targeting PSs like MB can inactivate ATP-dependent transporters, thereby inhibiting drug efflux. In addition, MB has been demonstrated to impair anti-apoptotic proteins such as BCL-2 and BCL-xL in mammalian cells [113,114]. Although these specific proteins have not been described in *Leishmania* spp., the presence of functionally analogous structures has been reported, opening new perspectives for exploring potential mechanisms of action [100,102,115,116].

Cabral et al. [71,93] demonstrated that DMMB-mediated PDT effectively eliminated both wild-type and miltefosine-resistant *L. amazonensis* strains. Our findings indicate that promastigotes exposed to MB, NMB, NMB-B, and NMB-P exhibited a more pronounced decline in viability compared to miltefosine. No evidence of resistant populations was observed, reinforcing the need for further investigations into their potential role in preventing resistance selection.

## 5. Conclusions

The results demonstrate for the first time the promising potential of the novel NMB derivatives, NMB-B and NMB-P, as PSs for PDT against *L. amazonensis*. PDT significantly enhanced the leishmanicidal activity of all the tested dyes, supporting our initial hypothesis. Inhibitory concentration values were markedly reduced by up to 85% under PDT conditions, with sustained effects resulting in lower viability compared to miltefosine-treated parasites. Remarkably, NMB-P achieved submicromolar potency. Moreover, our findings indicate that MB-mediated PDT induces photodamage in *Leishmania* through the generation of ROS, leading to apoptosis-like cell death with pronounced structural injury—a profile similarly exhibited by NMB and its derivatives.

To further elucidate the mechanism of action of the tested dyes under PDT against *L. amazonensis*, additional studies investigating mitochondrial function, parasite ultrastructure, and efficacy against the clinically relevant amastigote form are underway. In addition, exploring drug combinations with PDT may enhance treatment selectivity, mitigating toxicity and resistance. Taken together, our results highlight the therapeutic potential of NMB-based dyes in PDT as a promising antileishmanial strategy, warranting in vivo validation. Ultimately, given its topical applicability, reduced systemic toxicity, and potential cost-effectiveness, PDT may offer a feasible alternative to current chemotherapies. This is particularly relevant for impoverished populations, who are most affected by CL.

## Figures and Tables

**Table 1:** In vitro activity of methylene blue (MB), new methylene blue (NMB), new methylene blue B (NMB-B), and new methylene blue P (NMB-P) in the presence or absence of photodynamic therapy (PDT) with red light-emitting diode (LED) irradiation (300 s, 5.78 × 10^−4^ J/cm^2^) against promastigote forms of the *Leishmania amazonensis* PH8 strain (10^6^ parasites/mL) at 25%, 50%, and 75% inhibition concentrations (IC_25, 50, 75_) after 24 h of incubation. Miltefosine was used as the positive control.

Compounds	Antipromastigote Activity (μM)					
	IC_25_		IC_50_		IC_75_	
	PDT−	PDT+	PDT−	PDT+	PDT−	PDT+
MB	9.39 ± 2.03	2.73 ± 2.25 ^#,‡‡^	61.44 ± 4.41 ^d^	29.33 ± 4.31 ^##^	128.25 ± 0.07	106.55 ± 0.48 ^##^
NMB	2.34 ± 0.07	2.50 ± 1.75 ^‡‡^	5.42 ± 0.81 ^d^	5.28 ± 2.19 ^‡^	35.21 ± 1.85	11.04 ± 0.04 ^##,‡‡^
NMB-B	3.73 ± 3.20	1.56 ± 0.81 ^‡‡^	5.48 ± 1.73 ^d^	3.48 ± 0.99 ^‡^	71.32 ± 3.56	10.70 ± 0.58 ^##,‡‡^
NMB-P	1.50 ± 0.28	0.73 ± 0.16 ^#,‡‡‡^	2.84 ± 0.80 ^d^	2.48 ± 0.04 ^‡^	37.76 ± 8.91	7.20 ± 0.68 ^#,‡‡‡^
Miltefosine	13.03 ± 1.82		23.30 ± 2.55 ^d^		42.43 ± 4.86	

PDT− = IC values without PDT; PDT+ = IC values with PDT. d = IC_50_ values previously described [69]. Data are presented as mean ± SD. (#) *p* < 0.05; (##) *p* < 0.001 when comparing IC_25_, _50_, _75_ values of compounds with and without PDT by unpaired *t*-test. (‡) *p* < 0.05; (‡‡) *p* < 0.001; (‡‡‡) *p* < 0.0001 when comparing IC_25, 50, 75_ values of tested dyes with miltefosine.

**Table 2:** Flow cytometry analysis of reactive oxygen species (ROS) production on *Leishmania amazonensis* promastigote forms (5 × 10^6^ parasites/mL, PH8 strain) exposed to IC_50_ of methylene blue (MB), new methylene blue (NMB), new methylene blue B (NMB-B), and new methylene blue P (NMB-P), followed by photodynamic therapy (PDT) with red light-emitting diode (LED) irradiation (300 s, 5.78 × 10^−4^ J/cm^2^), 24 h of incubation, and labeling with H_2_DCFDA.

Compounds	Conc. µM	Low Population		High Population	
		MFI_H2DCFDA_	*VI*_ROS_	MFI_H2DCFDA_	*VI*_ROS_
MB	61.44	34,923.10	1.30 ± 0.59	1,084,378.70 *	2.43 ± 0.63
NMB	5.42	20,513.35	0.78 ± 0.55	2,016,352.20 **	4.49 ± 0.53
NMB-B	5.48	78,465.10	2.73 ± 1.31	1,781,042.90 **	4.00 ± 1.00
NMB-P	2.84	69,333.50	2.39 ± 1.48	2,118,590.15 **	4.67 ± 0.13
AA	10.00	41,057.05	1.50 ± 0.31	897,705.20	1.99 ± 0.16
LED-only	-	21,828.05	0.82 ± 0.40	413,811.30	0.90 ± 0.22
Control	-	27,786.50	-	452,851.90	-

IC_50_ = 50% maximal inhibitory concentration. Conc. = compound concentration. MFI = median fluorescence intensity. H_2_DCFDA = 2′,7′-dichlorodihydrofluorescein diacetate. MFI_H2DCFDA_ = median fluorescence intensity of the parasite labeled with H_2_DCFDA. *VI*_ROS_ (variation index of reactive oxygen species) = MT_H2DCFDA_/MC_H2DCFDA_, where MT corresponds to the H_2_DCFDA MFI of treated parasites, and MC corresponds to the H_2_DCFDA MFI of control parasites. AA = antimycin A. LED-only = untreated parasites exposed to 300 s of red LED irradiation (5.78 × 10^−4^ J/cm^2^) labeled with H_2_DCFDA. Control = untreated parasites labeled with H_2_DCFDA. Data are presented as mean ± SD. (*) *p* < 0.05; (**) *p* < 0.001 when compared to control by one-way ANOVA and Dunnett’s post-test.

## Data Availability

Data are contained within the article.

## Abbreviations

The following abbreviations are used in this manuscript:

AA	Antimycin A
AV	Annexin V
CL	Cutaneous leishmaniasis
DAPI	4′,6-Diamidino-2-phenylindole
DMBB	1,9-Dimethyl-methylene blue
FBS	Fetal bovine serum
H_2_DCFDA	2,7-Dichlorodihydrofluorescein diacetate
HP	High population
IC_25_	25% Inhibitory concentration
IC_50_	50% Maximal inhibitory concentration
IC_75_	75% Inhibitory concentration
LED	Light-emitting diode
LP	Low population
MB	Methylene blue
MFI	Median fluorescence intensity
NMB	New methylene blue
NMB-B	New methylene blue B
NMB-P	New methylene blue P
PBS	Phosphate-buffered saline
PDT	Photodynamic therapy
PI	Propidium iodide
PS	Photosensitizer
ROS	Reactive oxygen species
Sb(III)	Trivalent antimony
Sb(V)	Pentavalent antimonials
VI_ROS_	Variation index of ROS production
